# Multiple environmental cues impact habitat choice during nocturnal homing of specialized reef shrimp

**DOI:** 10.1093/beheco/ary171

**Published:** 2018-12-15

**Authors:** Molly M Ashur, Danielle L Dixson

**Affiliations:** School of Marine Science and Policy, University of Delaware, Lewes, USA

**Keywords:** coral reef, habitat selection, homing, *Lysmata pederseni*, peppermint shrimp, sensory systems, sponges

## Abstract

Habitat selection is a critical process for animals throughout their life, and adult organisms that travel to forage or mate must reselect habitat frequently. On coral reefs, competition for space has led to a high proportion of habitat specialists. Habitat selection is especially vital for organisms that require specialized habitat; however, research has primarily focused on the initial habitat choice made during the larval/juvenile stage. Here, we analyze habitat selection in the adult sponge-dwelling reef shrimp, *Lysmata pederseni*. Using a mark-and-recapture technique, belt transects, patch reefs, and cue isolation experiments, this study reveals that adult *L. pederseni* diurnally reselect habitat and a natural preference exists for specific sponge species and shapes. This natural preference is a function of chemical and morphological cues as well as sponge distribution. As habitat specialists can drive biodiversity, understanding the mechanisms behind habitat selection can inform research and management practices.

## Teaser

Is architecture more important than smell when choosing a home? Here, we investigate the external cues involved in habitat selection of a sponge-associated shrimp after nightly forays on the reef. By tracking shrimp locations through time and isolating environmental variables, we conclude that while both sponge shape and chemical cues play a role in habitat selection, shape may be a more critical factor for resident shrimp.

## INTRODUCTION

Animals typically occupy distinct habitats ranging in specificity from the entire ecosystem to specialized single-host microhabitats. Habitat selection has been researched in a variety of taxa in an attempt to understand the balance between environmental variables (Cowen and Sponaugle 2009) and animal behavior (Meadows and Campbell 1972). While some organisms choose appropriate habitat once during their life history—typically at the juvenile or larval stage—others must reselect habitat frequently if they migrate, disperse, or temporally home. Roaming animals exist in both the terrestrial environment (e.g., cougars [Dickson and Beier 2002], golden eagles [Domenech et al. 2015], and foxes [Chamberlain and Leopold 2000]) and the aquatic realm (e.g., catfish [Kadye and Booth 2012], octopuses [Regueira et al. 2013, and cardinalfishes [Marnane 2000]). Though these animals migrate frequently, they often have home bases and exhibit high site fidelity. Insight into adult habitat selection at all scales is critical as the ecological baselines of numerous systems are rapidly shifting. This shift is projected to most devastatingly influence species that are tightly coupled with specific habitats (Munday 2004).

Specialized habitat selection is particularly important in the intensely complex and diverse coral reef ecosystem (Huston 1985). The selection of favorable habitat can increase overall fitness through predator avoidance (Caley and St John 1996), food availability (Pereira et al. 2012), and access to mating opportunities (Baeza et al. 2016). Due to competition for space and the potential consequences of selecting inappropriate habitat (e.g., death), natural selection should favor individuals that choose suitable sites. On coral reefs, this selection has resulted in a high proportion of species exhibiting obligate habitat use (Connell 1978).

Obligate habitat associations often begin at the conclusion of the larval stage. Thereafter, the inhabitant rarely leaves the host, as seen in the anemonefish—sea anemone relationship (Dixson et al. 2014). However, some reef animals occupy specific habitats during rest periods, yet vacate to forage or mate. Cardinalfishes (Apogonidae), for example, reside in caves or dendritic corals during the day (Greenfield and Johnson 1990) but leave at night to forage throughout the reef (Chave 1978). Host specificity in cardinalfishes is pronounced, with individuals showing high site fidelity by homing back to specific locations within a coral matrix after being displaced 1–2 km (Marnane 2000). Such temporal homing has been observed in multiple species of reef teleosts (Ogden & Buckman 1973; Quinn and Brodeur 1991), as well as several invertebrate groups including crustaceans (Herrnkind and McLean 1971; Hahn and Itzkowitz 1986; Vannini and Cannicci 1995), cephalopods (Mather 1991), and limpets (Cook et al. 1969), yet the mechanisms behind the selection of habitat are relatively unknown.

The peppermint shrimp, *Lysmata pederseni*, exhibit obligatory associations with tube sponges, particularly the sponge genera *Callyspongia*, *Niphates*, and *Aplysina* throughout Caribbean reefs (Rhyne and Lin 2006; Baeza 2010; Baeza et al. 2016). Shrimp associations with tube sponges are rare among the family, *Hippolytidae*, which consists primarily of cleaner and peppermint shrimp (Rhyne and Lin 2006). Although *L. pederseni* is not a true cleaner shrimp, it may passively clean its surroundings (Rhyne and Lin 2006), potentially contributing to sponge health. Further, these cathemeral shrimp are ecologically distinct from other peppermint shrimp due to their propensity for living alone or in small groups, rather than gregariously (Rhyne and Lin 2006). Although *Lysmata sp.* are common within the aquarium trade for their ability to control the aquarium pest, *Aiptasia sp.* (Rhyne et al. 2004), little is known about the external factors that influence the unique habitat association of *L. pederseni* within tube sponges.

The evaluation of potential hosts can be accomplished through chemosensory perception or an assessment of morphological characteristics (Enright 1978). The relative use of these stimuli depends on species, spatial proximity, and environmental variables (Kingsford et al. 2002). *Lysmata pederseni* assess chemical cues using olfactory receptors in aesthetascs on their antennules (Hallberg et al. 1992). These shrimp are capable of detecting and responding to the chemical cues of *Callyspongia vaginalis* (Baeza et al. 2016); however, the role chemical cues play in species-specific habitat selection has yet to be understood. Additionally, host morphology can influence habitat choice, as size and shape can be critical not only for protection from predators, but also for space required to perform basic functions such as mating (Vytopil and Willis 2001). Morphological assessment can be accomplished using visual and tactile cues, making it a reliable source of information even for visually-limited shrimp (Caves et al. 2016).

In this study, we investigate the tube sponge habitat selection of adult *L. pederseni*. First, the movement patterns of adult peppermint shrimp were identified using a mark-and-recapture experiment. Then, belt transects were conducted to investigate natural variations in shrimp-associated sponge species and morphologies. Subsequently, to determine which sensory cues are pertinent for habitat selection, both chemical and morphological preferences were tested in isolation. Finally, as the natural distribution of sponge species varies spatially, patch reefs were constructed to test sponge preference when all cues were present and sponges were equidistant. Habitat specialists are more susceptible to the shifting species composition on coral reefs, especially when associated with living hosts. A better understanding of vital processes, such as specialized habitat selection, can lead to more thorough predictions of how ecosystems and communities will respond to future conditions. We use *L. pederseni* as a model organism in this study but the results have the potential to inform habitat selection across diverse taxa.

## METHODS

### Temporal movements

*Lysmata pederseni* habitat fidelity was monitored during the day and night to gain insight into the extent and temporal scale of adult movement and habitat selection. Short-term habitat fidelity was assessed using an overnight mark-and-recapture technique at 2 reefs near Carrie Bow Cay, Belize (16°48′9.26′′N, 88° 4′54.87′′W; fore reef: 17 m max depth, surveyed 23–25 August 2016; lagoon reef: 9 m max depth, surveyed 17–18 June 2017 and 24–27 June 2017). Sponges containing *L. pederseni* were marked with flagging tape in the afternoon (~16:00). If multiple sponge tubes hosted shrimp, each tube was individually flagged. Each shrimp within the sponge was carefully extracted by squeezing the sponge so that the shrimp rose through the column into a net (Baeza et al. 2016) and tagged using an elastomer tag (Northwest Marine Technology, Inc., USA) on the ventral side of the sixth abdominal segment (Baeza 2010). After tagging, shrimp were returned to their original sponge tube. All shrimp were alive after release (*n* = 26).

Shrimp locations were assessed 3 times following tagging. First, presence of shrimp was recorded the morning after the tagging procedure (~10:00) to appraise site fidelity. The subsequent night, shrimp-sponge associations were evaluated using a 1000 lumen flashlight at ~21:00 to determine whether shrimp nocturnally vacate their host sponge. Shrimp could be detected in a few seconds, which minimized disturbance by dive lights. Finally, flagged sponges were checked for shrimp presence the morning after the nocturnal observations (~10:00) to confirm site fidelity despite disturbance.

### Natural habitat associations

Natural variability in shrimp-sponge associations was determined using 15x1 m belt transects on reefs around Carrie Bow Cay, Belize between 31 March and 2 April 2016 (depth range: 3–14 m, *n* = 22). Along each transect, all tube sponges were inspected to quantify abundance of resident *L. pederseni* and 4 sponge parameters were recorded: 1) sponge species, 2) number of tubes, 3) tube height from base to tip, and 4) osculum diameter, measured at the widest point.

### Morphological preference

**Figure 1:**
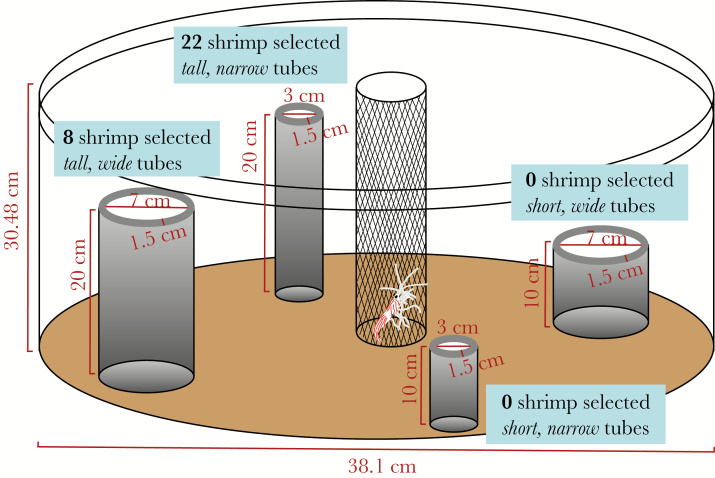
Infogram of the experimental methods to test sponge morphology preferences in isolation. Gray cylinders represent artificial sponges made of foam pool noodles. Experimental results are shown in text boxes with a significant preference for tall and narrow tube morphology.

Shrimp used in the cue isolation and patch reef experiments were collected using methods described above and transported to the Smithsonian Research Station on Carrie Bow Cay, where they were held in ~15 L flow-through aquaria with coral rubble for shelter until testing. Cafeteria-style choice experiments were used to assess shrimp morphological preference to host sponges. Four morphological types of artificial tube sponges were constructed from black foam pool noodles (Figure 1): tall wide (20 cm high, 7 cm osculum diameter), short wide (10 cm high, 7 cm osculum diameter), tall narrow (20 cm high, 3 cm osculum diameter), and short narrow (10 cm high, 3 cm osculum diameter). One of each sponge morphotype was fixed in a random configuration to an acrylic plate such that each artificial sponge was 30 cm from the center. The acrylic plates with attached artificial sponges were placed into circular aquaria (40 L) and weighed down with dive weights. Each aquarium was filled with seawater and sand was added to eliminate all crevices. A 10 cm diameter habituation chamber, constructed from plastic mesh (1.27 × 1.27 cm) and fly screen (1 × 1 mm), was positioned upright in the sand such that the top of the chamber was above the waterline. Each aquarium contained an airstone. The sand and water were replaced after each trial.

After sunset (~21:00), one shrimp was carefully placed inside the habituation chamber for 10 min. At the end of the habituation period, the chamber was slowly removed and the aquarium was covered with a mesh screen. This allowed natural light to penetrate but prevented the shrimp from escaping. After 12 h, the shrimp location was recorded (*n* = 36).

### Chemical preference

To assess the use of chemical cues in habitat selection, shrimp preference was tested using an Atema 2-channel choice flume (Gerlach et al. 2007). The choice flume (23.5 × 4 cm) allows an individual organism to experience the chemical cues of 2 different water sources simultaneously by presenting them side-by-side. The 2 water sources were gravity-fed from buckets through tubes into the choice chamber. Laminar flow was maintained at 100 mL min^−1^ using flow meters (Dwyer MMA-40) and checked periodically using dye tests. A shrimp was placed in the center of the flume and given a 2-min habituation period, during which time the shrimp was free to move throughout the chamber and explore either cue. After the habituation period, the shrimp’s position in each stimulus was recorded at 5-s intervals for 2 min. The water sources were then switched to eliminate a potential side bias with a 2-min flushing period and the entire test was repeated. Each shrimp was tested only once per trial (*n* = 10) and all trials were run blind.

Sponge species used as cues in fluming trials were chosen based on habitat associations observed during the transects (Table 1). *Lysmata pederseni* associated with the tube sponges, *Callyspongia vaginalis*, *Callyspongia plicifera*, and *Niphates digitalis*. In contrast, another common tube sponge, *Aplysinia fistularis*, never hosted peppermint shrimp and was used as a negative control cue. Sponges used for cue generation were cut at the base of their structures and transported to the lab. Only sponges lacking resident shrimp were collected and any additional epibionts were removed. To generate chemical cues, each species of sponge was spun 20 times in a salad spinner to eliminate excess water before being weighed to 20 g and added to 2 L of reef water for 1 h in a closed system. This solution was diluted to 10 L using reef water. Water collected directly from the reef acted as a general reef water cue. Chemical preferences were analyzed using the Kolmogorov–Smirnov (K–S) nonparametric test. The proportion of time spent in each cue was compared with the proportion of time spent on one side of the choice flume when no cues were present (blank control).

**Table 1:** Chemical choice comparisons

**Choice trials**	**Preference hierarchy trials**
A. fistularis* vs. Reef water	*C. vaginalis* vs. A. fistularis*
*C. vaginalis* vs. Reef water	*C. vaginalis* vs. *C. plicifera*
*C. plicifera* vs. Reef water	*C. vaginalis* vs. *N. digitalis*
*N. digitalis* vs. Reef water	*C. plicifera* vs. *N. digitalis*
Reef water vs. Reef water	Reef water vs. Reef water

* indicates nonassociated habitat based on transect data.

### Patch reefs

**Figure 2:**
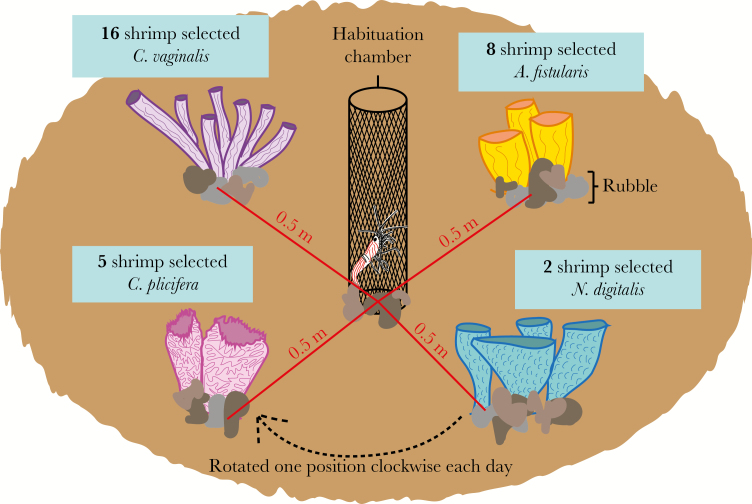
Infogram of the experimental methods to test sponge preference when all cues were present and sponges were equidistant. Experimental results are shown in text boxes with a significant preference for *C. vaginalis.*

The patch reef experiment was conducted in a large sand patch (8m depth, 16°48′45.15′′N, 88° 5′8.29′′W) to test the preference for different sponge species when all cues were present and sponge distribution was equal (Figure 2). Eight patch reefs were constructed and each contained one sponge of each of the 4 test species, *C. vaginalis*, *C. plicifera*, *N. digitalis*, and *A. fistularis*. Sponges were arranged randomly and size matched based on height and overall mass, as mass can influence the quantity of chemical cues released. Number of tubes and tube width were distinct morphological characteristics typical of specific sponge species and were therefore not size matched. Two rubble pieces were added to the center of the patch and each sponge was 0.5 m from this point and equidistance from each other.

Experimental shrimp were tagged with an elastomer tag to indicate the sponge species from which it was collected. Shrimp were placed on patch reefs at ~21:00 (*n* = 36). Dive lights never directly illuminated the patch reefs. At each patch, a 10 cm habituation chamber was wedged into the sand between the rubble pieces and one shrimp was carefully placed within this chamber. The chamber was open at the top, allowing the shrimp to leave and explore. After 30 min, the habituation chambers were removed. If the shrimp was still inside, the sides of the chamber were gently squeezed until the shrimp crawled onto the rubble pieces. After a 12-h overnight period, shrimp location was recorded and shrimp were removed from the patch reef. The sponges were shifted one position clockwise each morning to eliminate potential biases due to current direction or light availability. The experiment was conducted over 6 nights.

## RESULTS

### Temporal movements

Morning observations following afternoon tagging revealed that 96.2% ± 7.3 of tagged shrimp were found in the same sponge individual, and only 8.0% ± 10.6 of those found were within a different tube in the same sponge. Nocturnal observations conducted the subsequent night demonstrated definite movement from host sponges; 80.8% ± 15.1 of tagged shrimp vacated their host sponge entirely, with an additional 11.5% ± 12.3 migrating between different tubes of the same sponge. The majority of the tagged shrimp were absent from within or around the sponge individual; however, 5 tagged shrimp were observed outside of their host sponge tubes over the course of the experiment. Multiple untagged shrimp that were not present during the day were observed in flagged sponges at night. The morning following the nocturnal observations, 80.8% ± 15.1 of the tagged shrimp were found in the same sponge that they were originally tagged in, and only 23.8% ± 18.2 of those were found in a different tube within the same sponge. The proportion of shrimp present in their host sponges during the day compared to the night was statistically different (z-test for proportions in R [R Core Team 2017]; *P* < 0.0001), suggesting that homing is occurring nightly.

### Natural habitat associations

**Figure 3:**
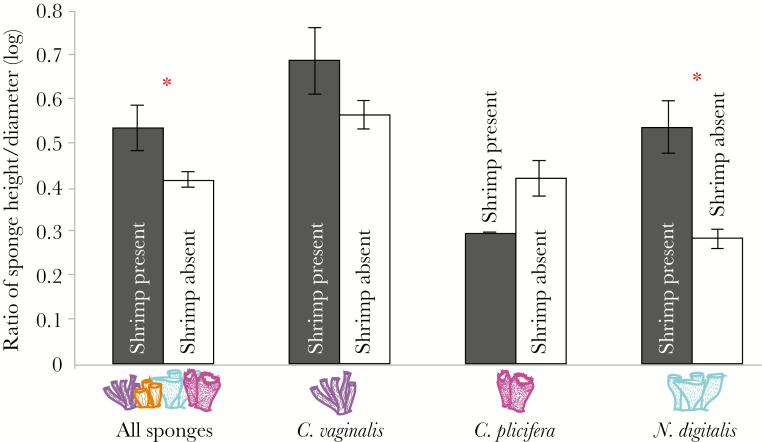
Natural shrimp-sponge associations. Transect data reveals a preference for tall narrow sponge tubes when all sponges are combined and when comparing *C. vaginalis* alone. White bars represent sponge tubes that did not host shrimp while gray bars represent sponge tubes with resident shrimp. * indicates significance.

Transects confirmed the natural association of *L. pederseni* to specific tube sponge species (See Supplementary Data). The number of tubes per sponge significantly influenced shrimp occupancy rates (analysis of variance [ANOVA] [JMP Pro 13]; *F*_7,148_ = 56.956, *P* < 0.0001) and among naturally shrimp-associated sponges, both sponge species (ANOVA; *F*_2,102_ = 4.8863, *P* = 0.0094) and number of tubes (ANOVA; *F*_6,102_ = 99.64, *P* < 0.0001) affected shrimp presence. The number of tubes, however, was highly correlated to the sponge species (ANOVA; *F*_9,148_ = 7.056; *P* = <0.0001). When sponge tube morphology was analyzed independent of sponge species, *L. pederseni* were more abundant on sponges with high height to diameter ratios (Wilcoxon signed-rank test [JMP Pro 13]; *P* = 0.0477, *n* = 234), thereby favoring tall, narrow sponges opposed to short, wide sponges (Figure 3). An analysis of individual species morphologies reveals that *L. pederseni* tend to occupy *N. digitalis* tubes with high height to diameter ratios (Wilcoxon signed-rank test; *P* = 0.0012, *n* = 89). No morphological inclinations were observed for *C. vaginalis* (Wilcoxon signed-rank test; *P* = 0.1605, *n* = 16) or *C. plicifera* (Wilcoxon signed-rank test; *P* = 0.4768, *n* = 42). The natural abundance of sponge species varies spatially; when the relative population abundance of each sponge species was considered, a greater percentage of *C. vaginalis* tubes hosted *L. pederseni* (18.8%, *n* = 16), followed by *N. digitalis* (8.9%, *n* = 89) and lastly *C. plicifera* (4.8%, *n* = 42). Additionally, tube sponges hosted other epibionts including fish, crabs, brittle stars, and nonconspecific shrimp, but the presence of other inhabitants did not preclude the focal shrimp from association with sponge individuals.

### Morphological preference

When morphological preferences were tested in isolation using artificial sponges, no shrimp were found in association with short sponges, regardless of osculum width (Figure 1). Shrimp significantly preferred taller (*χ*^*2*^ goodness-of-fit test in [R Core Team 2017]; *χ*^*2*^ = 28.033, *P* < 0.0001) and narrower (*χ*^*2*^ goodness-of-fit test; *χ*^*2*^ = 5.633, *P* = 0.0176) tubes. Of the 36 shrimp tested, 6 did not choose an artificial sponge, 22 selected tall, narrow sponges, and 8 selected tall, wide sponges.

### Chemical preference

**Figure 4:**
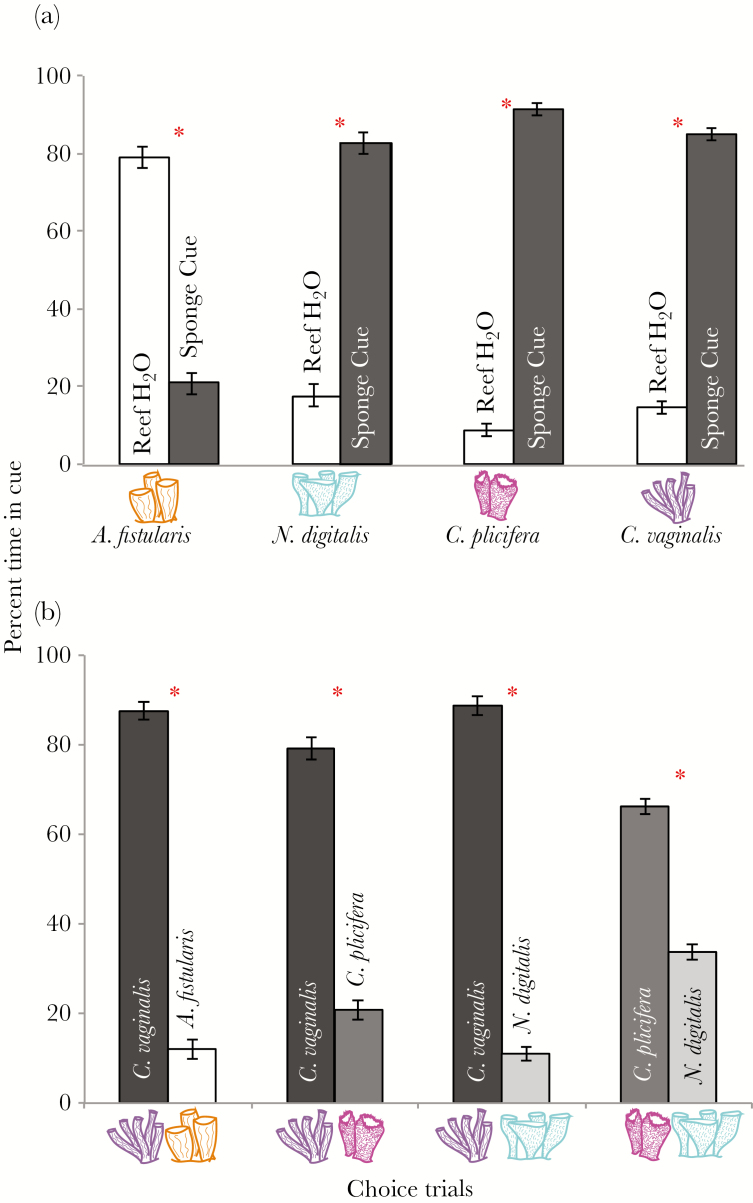
Chemical choice trials. (A) Shrimp response to individual sponge cues compared to general reef cues. (B) Shrimp response when presented with 2 sponge cues simultaneously to determine a hierarchical preference. All chemical data are mean percent time ± standard error. * indicates significance.

Shrimp preferred the chemical cues of *C. vaginalis*, *C. plicifera*, and *N. digitalis* to the general reef cue, by spending >80% of their time in the side of the flume containing the sponge cue (K–S test [JMP Pro 13]; *P* < 0.0001). However, *L. pederseni* avoided the chemical cue from the negative control, *A. fistularis*, only spending 21% ± 2.66 SE of the time in this cue (K–S test, *P* < 0.0001) (Figure 4a). When presented with 2 different sponge cues simultaneously, peppermint shrimp preferred *C. vaginalis* to all other species tested (K–S test; *P* < 0.0001 for either comparison). When chemical cues from *C. plicifera* were tested against the chemical cues of *N. digitalis*, shrimp preferred *C. plicifera* (K–S test; *P* < 0.0001) (Figure 4b).

### Patch reefs

The sponge species that shrimp were collected from did not impact sponge choice in the patch reef experiment (Fisher’s Exact test in R [34]; *P* = 0.3512), allowing data from all of the shrimp to be pooled. Sponge selection by *L. pederseni* was not random (*χ*^*2*^ goodness-of-fit test in [R Core Team 2017]; *χ*^*2*^ = 14.032, *P* = 0.002862); rather, *L. pederseni* significantly preferred *C. vaginalis*, with 51% (*n* = 16) of shrimp associating with this species the following morning. *C. plicifera* was selected by 16% (*n* = 5) of the shrimp and 6% (*n* = 2) of the shrimp selected *N. digitalis* (Figure 2). Surprisingly, 26% of the shrimp (*n* = 8) selected *A. fistularis*, an association never observed naturally. Five shrimp either abandoned the patch reef or succumbed to predation.

## DISCUSSION

As the shifting climate causes changes in local species abundances (Hughes et al. 2003), habitat specialists will be some of the most vulnerable due to their association with living hosts (Munday 2004). Understanding specialized habitat associations and the mechanisms used in assessing host individuals is vital at both the larval and adult stages. Here, we show that adult *L. pederseni* vacate and reselect habitat daily, with strong site fidelity towards the same host individual. These shrimp demonstrate a preference for the chemical cues of specific sponge species as well as the morphological characteristics of tall narrow sponge tubes, a pattern that is reflected in both natural benthic transects and the in situ patch reef experiment. The natural distribution of sponges, however, can also influence sponge-shrimp associations across the reef, thereby creating high variation in chosen host sponges.

Most obligate habitat associations begin at the larval stage and thereafter the inhabitant remains solely within the host. In contrast, *L. pederseni* show high site fidelity to individual sponges, yet vacate at night, suggesting that adult habitat reselection is frequent. Previous studies have indicated that *L. pederseni* host fidelity can be constant for up to 2 months (Baeza 2010), depending on the sexual phase or mating system (Baeza et al. 2016). *Lysmata pederseni* are protandric simultaneous hermaphrodites, where all juveniles are male and develop into functional hermaphrodites (Baeza 2009). Populations can tend towards monogamy or polygynandry depending on location. Populations at Carrie Bow Cay, Belize are primarily monogamous (Baeza et al. 2016), whereas populations in the Florida Keys exist in polygynandrous relationships characterized by promiscuity and frequent host switching (Baeza 2010). Previous studies, however, have only assessed site fidelity during the day. Our study confirms that regular nocturnal vacancies occur despite apparent daytime fidelity to specific sponges, suggesting that habitat selection is an essential process at the adult stage even among monogamous populations.

Morphological characteristics tested in isolation revealed a preference for tall, narrow sponges. This morphological affiliation is confirmed in field transects, but whether these natural associations are due to shrimp selection or differential survival remains to be determined. Specifically, morphological characteristics of *C. vaginalis* and *C. plicifera* do not influence the likelihood of shrimp association, possibly due to their ubiquitous tube-like shapes. Conversely, shrimp are more abundant on tall, narrow morphotypes of *N. digitalis*. Tube shape of *N. digitalis* varies with some having an irregularly shaped osculum that is long and narrow. Measurements were taken at the widest diameter of the osculum, causing irregular morphotypes to have low height to diameter ratios. Wider openings may allow easier predator access to the epifaunal community within and may promote shrimp to avoid this morphotype. Height preference in this species is further confirmed in the Florida Keys where *L. pederseni* were never found in sponges smaller than 10 cm high (Baeza et al. 2016). While our isolation experiment validates this, one shrimp was found naturally within a 6 cm *C. plicifera* tube.

*Lysmata pederseni* easily discriminated between different sponge odours. Chemical preferences (*C. vaginalis*, *C. plicifera*, and *N. digitalis*) and chemical deterrents (*A. fistularis*) matched natural presence-absence field observations. Previous work on the chemical detection of *L. pederseni* found that these shrimp positively respond to the chemical cues of *C. vaginalis* (Baeza et al. 2016). Our study corroborates and expands on this research by testing the response to additional host species and identifying hierarchical preferences. *Lysmata pederseni* have the strongest preference for the chemical cues of *C. vaginalis*, followed by *C. plicifera* and *N. digitalis*. This ranking is supported by previous field studies stating that *L. pederseni* are more commonly or only found in *C. vaginalis* (Rhyne and Lin 2006; Baeza 2010; Baeza et al. 2016).

Reef sponges can be classified into 3 categories in relation to chemical defenses and predation rates: 1) chemically-defended species, 2) chemically-undefended species that persist because of high rates of growth, reproduction or healing, and 3) chemically-undefended species that persist in secluded refuges (Pawlik 2011). *Callyspongia* and *Niphates* are fast-growing and chemically-undefended (Pawlik et al. 1995), whereas *Aplysina* sponges produce secondary metabolites that defend against predation (Pawlik et al. 1995), fouling (Willemsen 1994), microbial growth (Kelly et al. 2005), and allelopathic attacks (Pawlik et al. 2007). *Aplysina fistularis*, in particular, naturally extrudes secondary metabolites, but the rate of extrusion can increase when damaged (Walker et al. 1985). Transect and fluming data indicate that *L. pederseni* prefer the fast-growing, chemically-undefended species, suggesting that sponge morphology may play a larger role in shrimp preference than the chemical defense of the habitat itself.

The differential distribution of sponges across the reef could be a major factor contributing to sponge-shrimp associations. Transects revealed more *L. pederseni* residing in *N. digitalis* than other species; however, after factoring in sponge population sizes, peppermint shrimp inhabited a higher proportion of *C. vaginalis* sponges, matching the chemical preference data. When the influence of distance and scarcity was eliminated in the patch reef experiment, *L. pederseni* preferred *C. vaginalis*, regardless of origin sponge. Although natural sponge associations and chemical cue preferences corroborate one another, the patch reef data demonstrated the willingness of shrimp to associate with *A. fistularis*. This sponge never hosted a shrimp naturally and was avoided using chemical cues alone. The association of eight *L. pederseni* individuals with *A. fistularis* in the patch reefs indicates that the secondary metabolites produced by this sponge are not acutely toxic to the shrimp; however, as seen with other sponge-dwelling species, these secondary metabolites could have chronic effects (Henkel and Pawlik 2011). The growth rate of the brittle star, *Ophiothrix lineata*, was significantly reduced when experimentally forced to reside in a sponge species that produces secondary metabolites rather than in a preferred nondefended sponge (Henkel and Pawlik 2011). The selection of *A. fistularis* by peppermint shrimp in the patch reef also supports the idea that sponge morphology has a greater influence than sponge chemistry on short-term habitat selection. *Aplysina fistularis* had the highest height to diameter ratio of all tube sponges on the transects, potentially making this species a beneficial choice for a shrimp looking to quickly escape from predators. The absence of *L. pederseni* in *A. fistularis* sponges in nature suggests that despite beneficial morphology, the secondary metabolites produced by this sponge may have long-term consequences.

In conclusion, *L. pederseni* utilize morphology as an effective habitat selection tool and also show distinct chemical preferences for specific hosts. Habitat selection in adults is understudied compared with habitat selection in larvae, but is no less important as movements between distinct habitats can influence material transport, nutrient fluxes and community dynamics (Marnane 2000). Understanding how and why adult habitat specialists choose their particular habitat is important in today’s rapidly changing world. Habitat degradation is occurring at an unprecedented rate, especially in reef environments (De’ath et al. 2012). Habitat specialists may be more threatened by environmental change when they are associated with a living host because the host can also be impacted by changing conditions (Munday 2004). Since habitat specialists can drive biodiversity (Sale 1977), understanding the mechanisms behind habitat selection can inform research and management practices.

## FUNDING

Research was funded by the Gordon and Betty Moore Foundation through Grant GBMF5464 (D.L.D.), Alfred P. Sloan Foundation (D.L.D.) and NSF GRFP 1247394 (M.M.A.).

## Supplementary Material

Supplementary Data
