# Expression of Concern: Multiple environmental cues impact habitat choice during nocturnal homing of specialized reef shrimp

**DOI:** 10.1093/beheco/arac108

**Published:** 2022-12-01

This is an Expression of Concern regarding: Molly M Ashur, Danielle L Dixson, Multiple environmental cues impact habitat choice during nocturnal homing of specialized reef shrimp, *Behavioral Ecology*, Volume 30, Issue 2, March/April 2019, Pages 348–355, https://doi.org/10.1093/beheco/ary171

The Editor-in-Chief has been alerted to concerns about the credibility of the data in the above paper.

The journal is publishing this Expression of Concern to alert readers while we investigate to determine whether further action is required.

