# Supplementary material for: Multiple environmental cues impact habitat choice during nocturnal homing of specialized reef shrimp
Source: Behav Ecol. 2018 Dec 15;30(2):348–55. doi: 10.1093/beheco/ary171 (PMC6450203; doi:10.1093/beheco/ary171)
Supplement: Supplementary Data [file ary171_suppl_supplementary_data.docx]

Table S1: Natural variation in sponge distribution, morphology, and *Lysmata pederseni* association. Unknown A refers to an unidentifiable sponge species characterized by black coloration and rough exterior (image Appendix S2)

| **Species** | **# Sponges in transect** | **Mean**  **# tubes** | **Mean tube height** | **Mean tube diameter** | **Mean diameter/ height ratio** | **# Shrimp** |
| --- | --- | --- | --- | --- | --- | --- |
| ***Callyspongia vaginalis*** | 3 | 5.333 | 14.750 | 3.813 | 0.266 | 3 |
| ***Aplysina fistularis*** | 5 | 2.400 | 12.750 | 2.083 | 0.167 | 0 |
| ***Agelas tubulata*** | 5 | 4.200 | 12.952 | 3.190 | 0.252 | 0 |
| ***Callyspongia plicifera*** | 33 | 1.273 | 17.905 | 6.095 | 0.444 | 2 |
| ***Niphates digitalis*** | 67 | 1.328 | 18.000 | 9.584 | 0.550 | 8 |
| ***Mycale laxissima*** | 11 | 1.000 | 13.636 | 7.091 | 0.570 | 0 |
| ***Aplysina archeri*** | 2 | 4.000 | 12.250 | 3.500 | 0.290 | 0 |
| ***Neofibularia nolitangere*** | 2 | 1.000 | 14.500 | 11.000 | 0.762 | 0 |
| ***X. muta*** | 5 | 1.000 | 24.200 | 18.600 | 0.749 | 0 |
| ***Unknown A*** | 16 | 1.750 | 15.429 | 5.071 | 0.370 | 0 |


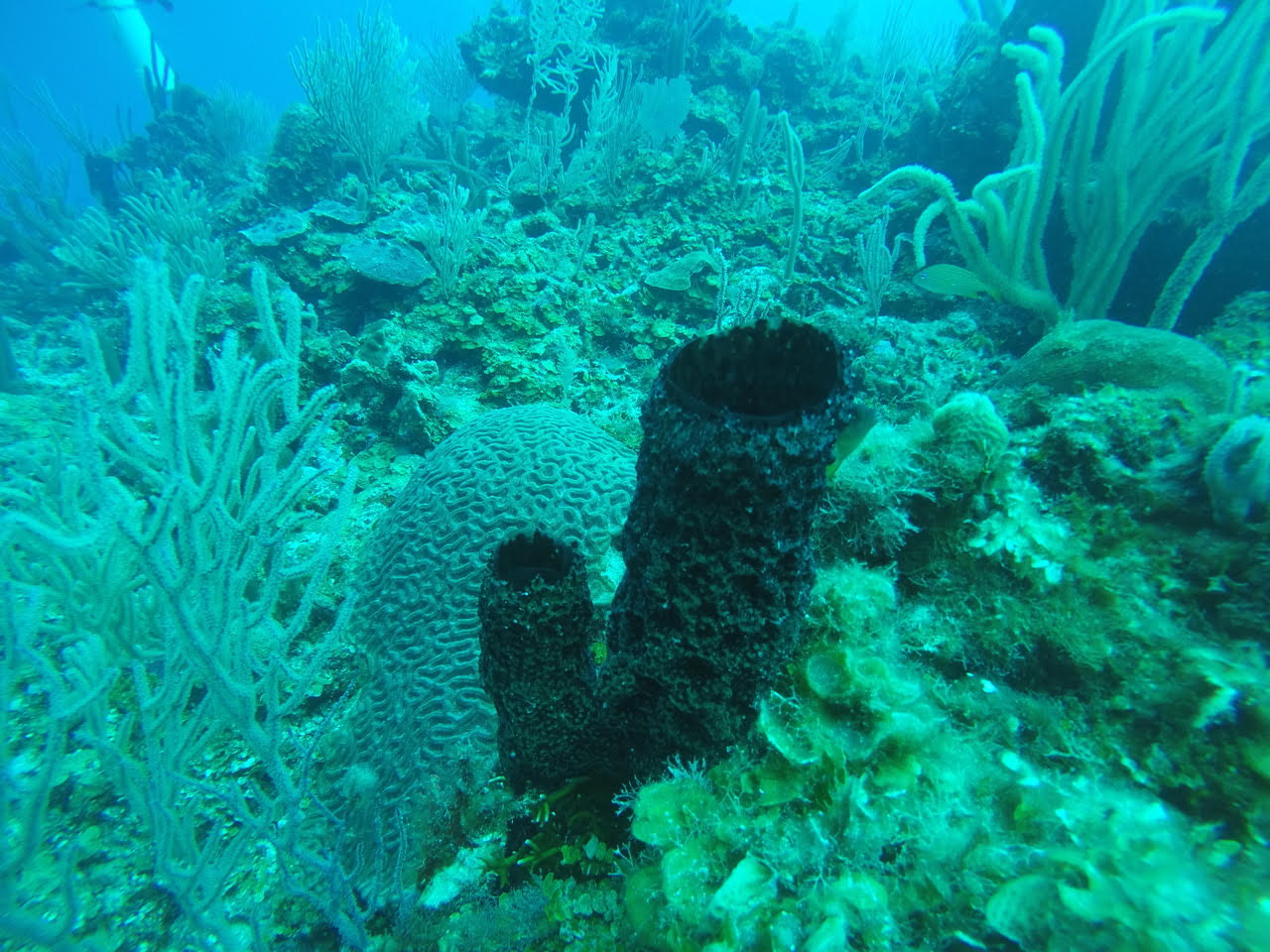


**Figure S1:** Photograph of unidentifiable Sponge A
